# Diquat Poisoning: Care Management and Medico-Legal Implications

**DOI:** 10.3390/toxics10040166

**Published:** 2022-03-30

**Authors:** Pascale Basilicata, Maria Pieri, Angela Simonelli, Emanuele Capasso, Claudia Casella, Tina Noto, Fabio Policino, Pierpaolo Di Lorenzo

**Affiliations:** 1 Department of Advanced Biomedical Science-Legal Medicine Section, University of Naples “Federico II”, 80138 Naples, Italy; pascale.basilicata@unina.it (P.B.); maria.pieri@unina.it (M.P.); emanuele.capasso@unina.it (E.C.); claudia.casella@unina.it (C.C.); fabio.policino@unina.it (F.P.); pierpaolo.dilorenzo@unina.it (P.D.L.); 2 Department of Forensic and Forensic Medicine, University of Murcia, 30100 Murcia, Spain; tina.noto@um.es

**Keywords:** diquat, suicidal ingestion, patient management, professional liabilities

## Abstract

Acute chemical intoxication represents one of the major causes of Emergency Room admittance, and possible errors in diagnosis are extremely frequent, especially when patients present generic and non-specific symptoms. Diquat, a bipyridyl class of herbicides, exerts high intrinsic toxicity as a consequence of free oxygen radicals, leading to cellular death and organ dysfunctions. Following ingestion, with the major source of absorption for suicidal purposes, the chemical induces local irritating effects; systemic symptoms appear later, while specific symptoms can occur in the following 48 h. A smoker and hypertensive 50-year-old man arrives at the E.R., reporting that an episode of herbicide inhalation occurred few hours earlier. Physical examination evidenced alkalosis with hypoxemia, leucocytosis, mild hyperglycaemia and moderate increase in creatine kinase and myoglobin. Despite blood creatine kinase and myoglobin values that were higher than normal, he was prescribed with hydration and anti-pain therapy. During the night, the man left the hospital; he returned the next morning at 8:45 a.m., with cardiorespiratory arrest, medium fixed non-reactive mydriasis, diffused cyanosis of the skin and of the mucous membranes, as well as imperceptible pulse and peripheral pressure. Despite resuscitation attempts, the patient died at 9:30 a.m.; the body was immediately transferred to the morgue. Autopsy and toxicological analyses were carried out nine days later, evidencing paraquat ingestion for suicidal purposes. GC/MS analyses to verify the presence of diquat were performed on body fluids and gastric and colon contents; all specimens resulted positive, thus confirming the cause of death as herbicide ingestion (blood diquat concentration of 1.2 mg/L; more than twice the minimum to observe a systemic poisoning). The procedure followed for patient management resulted to be not in line with the provisions of both guidelines and good clinical practices. Staff did not perform clinical-diagnostical monitoring of the patient’s condition or ask for more specific analyses (i.e., serum creatine phosphokinase monitoring). This misconduct led to a decrease in the patient’s chances to survive.

## 1. Introduction

Exposure to chemicals represents a major source of accidents and hospitalizations; applications for compensation may derive from incorrect patient management [1,2]. The XII edition of the MadMed survey report on errors related to medical malpractice in Italy between 2004 and 2019 showed that Orthopaedics and Traumatology Operative Units together with Emergency Rooms (E.R.; the principal unit involved in the management of patients showing chemical poisoning) present the highest frequency of adverse events (20.1% and 14.2%, respectively), followed by General Surgery Departments, showing 13.2% of adverse events [3]. In the E.R., errors can derive from incorrect diagnosis (59.9%) and therapies (25.2%), both resulting in severe consequences in terms of permanent disability or death; estimated costs related to each error amounts to about 92.547 euros [3].

Acute intoxication derives from a dynamic process characterized by a rapid negative evolution with possible lethal consequences, even when symptoms are initially mild; exposure to xenobiotics represents a major source of acute intoxication, and severity is generally dose-related [4]. Symptoms deriving from poisoning vary according to the chemical/physical characteristics of the xenobiotic involved. Absorption route, exposure time, interpersonal variability, and the subject’s general health condition also play a critical role in determining the specific effects registered in each patient. The times of evolution may differ, as the manifestations of toxicity can be delayed by the exposure time. Clinical manifestations are strictly related to chemical and physical properties of the substance and can be used to determine the duration of the exposure and the absorption routes. These include stomatitis, enteritis, or perforations of the gastrointestinal tract mucous membrane as a consequence of caustic/corrosive substance ingestion; halitosis, in the case of alcohol or hydrocarbon ingestion; erythema, pain, or blisters after dermal absorption (frequent in the case of accidents); lesions in the cornea, sclera, and lens, with eye pain, redness, and loss of vision associated with liquid spills. Anatomical localization of lesions can differ in accordance with solubility (inhalation of toxic water-soluble substances, i.e., chlorine or ammonia, can cause symptoms in the upper airways; otherwise, lesions may occur at the lower airway region or involve non-cardiogenic pulmonary oedema).

Diquat (6,7-dihydrodipyrido[1,2-a:2′,1′-c]pyrazine-5,8-diium dibromide) is a non-selective contact herbicide characterized by a high toxic capacity, commercialized as a paraquat substitute [5]. Treatment of poisoning due to this herbicide requires extensive experience. Clinical manifestations associated with diquat poisoning imply gastroenteritis and acute renal failure, but in severe cases it can lead to respiratory failure, cardiovascular collapse, arrhythmias, seizures, coma with cerebral haemorrhage, and heart attack [6]. Inhalation of the aerosol is generally associated with mild symptoms, rarely resulting in fatal outcomes [7,8]. Ingestion of high diquat doses for suicidal purposes, the most common cause of poisoning, may result in the subject’s death during the next one or two days, as reported for a man who ingested about 160 mL of enriched diquat (20 g per 100 mL) [9]. Diquat intoxication can lead to severe toxic effects on the central nervous system, with manifestations including nervousness, irritability, restlessness, aggression, disorientation, senseless reasoning, inability to recognize family or friends, and reduced reflexes. Neurological effects can progress to coma, accompanied by tonic-clonic seizures, and culminate in the patient’s death [5,10]. Moreover, diquat ingestion produces corrosive manifestations on digestive tract tissues, with the appearance of burnings in the mouth, throat, chest, and abdomen; intense nausea and vomiting and diarrhoea can appeal up to two days after exposure to low doses, and blood may appear in vomit and stool.

Once absorbed, diquat is excreted through the kidney, the target organ and a useful intoxication index for clinicians [5,6]. Proteinuria, haematuria, and pyuria can progress to renal failure and uraemia. Toxic effects can include liver, pancreatic, heart, and muscle damage; jaundice and liver lesions may arise as evidenced by an increase in alkaline phosphatase, transaminase, and LDH values. If the patient survives several hours or days, cardiocirculatory failure due to dehydration may occur, as well as hypotension and tachycardia, with shock progressing to death. The picture can evolve towards cardiorespiratory problems, including toxic cardiomyopathy, or a secondary infection, such as bronchopneumonia [11].

### Case Report

A smoker and hypertensive 50-year-old man came to the E.R. at 9:00 p.m., reporting an episode of accidental inhalation of herbicide happening few hours earlier. The toxic substance and duration of exposure time were not specified.

Physical and laboratory examination: 170/100 SBP, 97% SaO_2_; alkalosis with hypoxemia (ABG test: 7.55 pH, pCO_2_ 23 mmHg, pO_2_ 69 mmHg), leucocytosis (WBC 14.07 × 10^3^/µL—v.n. 4.5–10), mild hyperglycaemia (glucose 123 mg/dL—v.n. 60–110) and moderate increase in creatine kinase (307 UL/L—v.n. < 170) and myoglobin (145.6 ng/dL—25–72). Renal (urea 35 mg/dL—v.n. 15–50; creatinine 0.62 mg/dL—v.n. 0.6–1.3) and hepatic (AST 26 UI/L—v.n. < 40; ALT 30 UI/L—v.n. < 40; GGT 23 UI/L—v.n. 10–71) function parameters were normal. A chest contrast-enhanced CT (ECCT) evidenced blurred and diffuse centrilobular opacities of both upper lobes; an angio-ECCT scan of the abdomen and pelvis excluded internal organ lesions. The patient was hospitalized in the Short Stay Observation Unit at 11:00 p.m., because he suffered from burning in the lower limbs. Physicians prescribed hydration and pain relief therapy (paracetamol in 500 mL saline solution). Vital signs (blood pressure, heart rate, respiratory rate, body temperature, diuresis) were not monitored, nor was the onset of a more specific symptomatology. The next day physicians decided on discharge, but at 8:30 a.m. the man was not in his room. At 8:45 a.m. he arrived at the E.R. in critical condition, presenting cardiorespiratory arrest with medium fixed non-reactive mydriasis, diffuse cyanosis of the skin and mucous membranes, as well as imperceptible pulse and peripheral pressure.

Despite the attempts to resuscitate him, the patient died at 9:30 a.m., and the body was immediately transferred to the morgue. Both autopsy and toxicological analyses were performed nine days later, to clarify the exact cause of death (with particular attention to possible poisoning due to herbicide) as well as to verify eventual professional liabilities.

The identification of the toxic substance was made possible by toxicological examination of the liquids and the biological remains obtained during the autopsy.

There was no evidence that the patient took an additional dose of herbicide when he left the hospital; no further intake by family members was reported.

## 2. Material and Methods

Certified standard solutions of chemicals used for confirmatory analysis in gas chromatography/mass spectrometry (GC/MS) were from Cerilliant-Merck (Milan, Italy), *N*,*O*-bis(trimethylsilyl)trifluoroacetamide (BSTFA) derivatizing agent from Acros (Morris Plains, NJ, USA), and HPLC grade solvents from Carlo Erba (Milan, Italy). Solid phase extraction was made using Strata-C18.

Immunochemical screening tests were carried out on a Randox Evidence Investigator (Randox Toxicology, Country Antrim, UK), using DoA I + WB SQ and DoA II WB whole blood tests for AMP/MAMP/MDMA, barbiturates, benzodiazepines, buprenorphine, cannabinoids, cocaine, methadone, opiates, phencyclidine, tricyclic antidepressants, fentanyl, ketamine, LSD, methaqualone, oxycodone, and propoxyphene.

GC/MS analyses were performed using a DSQ single quadrupole mass spectrometer directly linked to a AS3000 gas chromatograph equipped with a *split-splitless* autosampler, all from ThermoFisher (San José, CA, USA). Gas chromatographic separations were made with a Rxi^®^-5MS (30 m × 0.25 mm × 0.25 µm) capillary column (Restek, Bellefonte, PA, USA). Data were processed using the Xcalibur software (version 2.0.7) from ThermoFisher.

Head-space gas chromatographic/mass spectrometric (HS-GC/MS) analyses were performed on an HP6890 series gas chromatographer provided with a HP7694E autosampler and a 5973 single quadrupole mass spectrometer (Hewlett-Packard, Palo Alto, CA, USA); chromatographic separation was accomplished by a CP PorabondQ capillary column (Varian, Crawley, UK), and data were analyzed using the MSD Chemstation software (D.02.0.275 version) from Agilent Technologies (Santa Clara, CA, USA).

### 2.1. Toxicological Analysis

Biological fluids (peripheral blood, urine, and bile) and gastric and colon contents (g.c. and c.c., respectively) were used for complete toxicological analyses. Screening tests were initially carried out on 100 µL aliquot of peripheral blood and processed according to immunoassay system specifications.

Specific conformation analyses were performed on all biological matrices by GC/MS, after proper purification through solid phase extraction and eventual derivatization [12,13,14]. For GC-MS analyses, all samples were acquired both in *full scan* and selected ion monitoring mode (GC/MS-SIM). Specific GC/MS analyses to verify the presence of diquat were done on body fluids, g.c., and c.c. At this stage, 1.5 mL aliquots of each biological sample were treated with 10 mg NaBH_4_ at 60 °C for 10 min to allow diquat reduction. Samples were subsequently purified by solid phase extraction. Cartridges were conditioned with 2 mL methanol and 2 mL phosphate buffer (pH 8); after sample loading, cartridges were washed with 2 mL bidistilled water, then dried for 5 min before elution with 2 mL methanol. Eluted samples were dried under nitrogen stream and then redissolved in 200 µL methanol for GC/MS *full scan* and SIM analyses.

The possible presence of ethyl alcohol or any other volatile chemical was also verified by analysing aliquot peripheral blood using HS-GC/MS.

### 2.2. Diquat Quantification

#### 2.2.1. Sample Preparation and Purification

A four-point standard addition protocol was used to quantify diquat (DQ) in blood, bile, urine, and gastric and colon contents using paraquat (PQ) as an internal standard (i.s.).

For each biological sample, four aliquots (1 mL for blood and urine; 0.5 mL for bile, gastric and colon contents) were analyzed. The specimens were added with 50 µL of a 20 ng/µL paraquat solution.

Standard addition samples were prepared as follows: “zero” point, biological matrix was spiked with i.s.; A–C samples, biological matrix was spiked with 50 µL of diquat solutions at concentrations of 80, 40, and 20 ng/µL, respectively, and corresponding to urine diquat concentrations of 4, 2, and 1 µg/mL in blood and urine and 8, 4, and 2 µg/mL in bile, gastric and colon contents, respectively.

Conversion of quaternary ammonium compounds, such as DQ and PQ, in thermally stable and volatile substances is essential for gas chromatographic analysis. The reaction is successfully carried out with sodium borohydride and applied to the gas chromatographic/mass spectrometric analysis of blood, urine, bile, and gastric and colon contents samples. Samples were treated with 10 mg of sodium borohydride (NaBH_4_) to reduce diquat into a more volatile compound. The reaction was conducted for 10 min at 60 °C. SPE extraction was performed with Strata-C18 E (200 mg/3 mL), involving drop-to-drop elution at 5 mmHg and the following extraction procedure: conditioning: 2 mL methanol and 2 mL phosphate buffer (pH 8); sample loading; washing: 2 mL bidistilled water; elution: 2 mL methanol. The eluted fraction, dried under nitrogen stream, was reconstituted in 100 µL methanol and 1 µL was injected into the GC/MS system, then analyzed according to de Almeida et al. [15].

#### 2.2.2. GC/MS Analysis and Quantification

The GC oven temperature was kept at 150 °C for 1 min; then, the temperature was increased up to 300 °C at 20 °C/min. Helium (purity: 99.5%) was used as carrier gas at 1 mL/min, with a constant flow mode. The MS detector (source temperature, 240 °C) operated in the selected ion monitoring (SIM) mode; acquired ions: *m/z* 108, 135, and 190 for diquat; *m/z* 134, 148, and 192 for paraquat.

The ratio of peak areas between diquat and paraquat was worked out and considered as the detector response. According to the standard addition approach [16], quantification was based on detector responses recorded for “zero point” and A–C spiked samples versus spiked analyte amount. A straight line was drawn, and the value of the x intercept represented the amount of the analyte in the unknown sample.

## 3. Results

### 3.1. Autopsy

**Figure 1:**
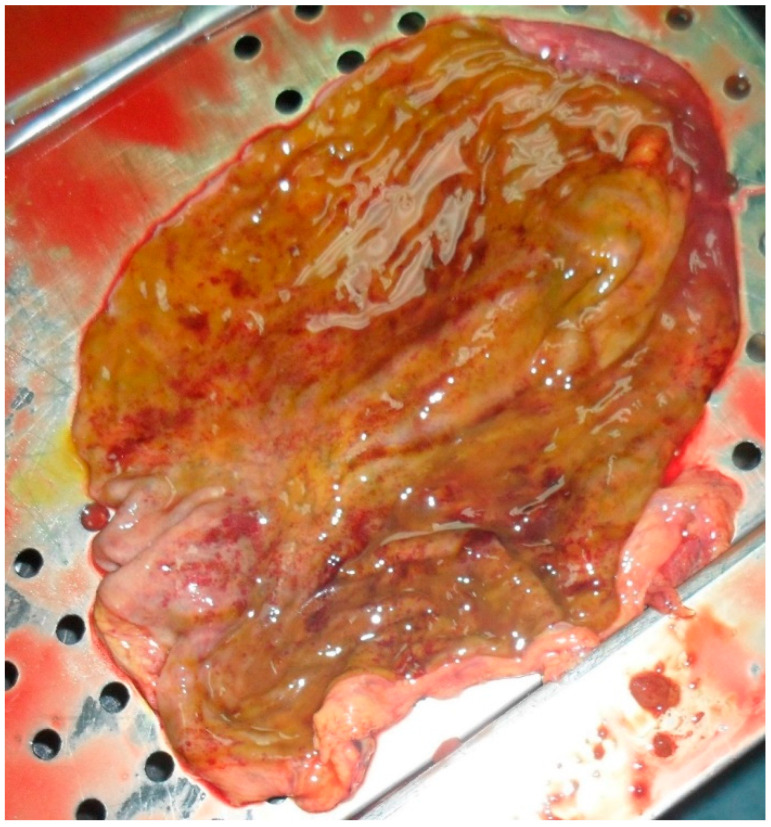
Internal stomach walls with areas of inflammation.

The autopsy evidenced the following: congestion of meningeal vessels; oedema and congestion of both lungs; left ventricular hypertrophy with widespread congestion and sclerosis of both valves and coronaries; inflammation of small intestine and stomach, both presenting a greenish liquid with a very intense smell (see Figure 1); congestion of spleen and kidneys.

### 3.2. Histological Analyses

**Figure 2:**
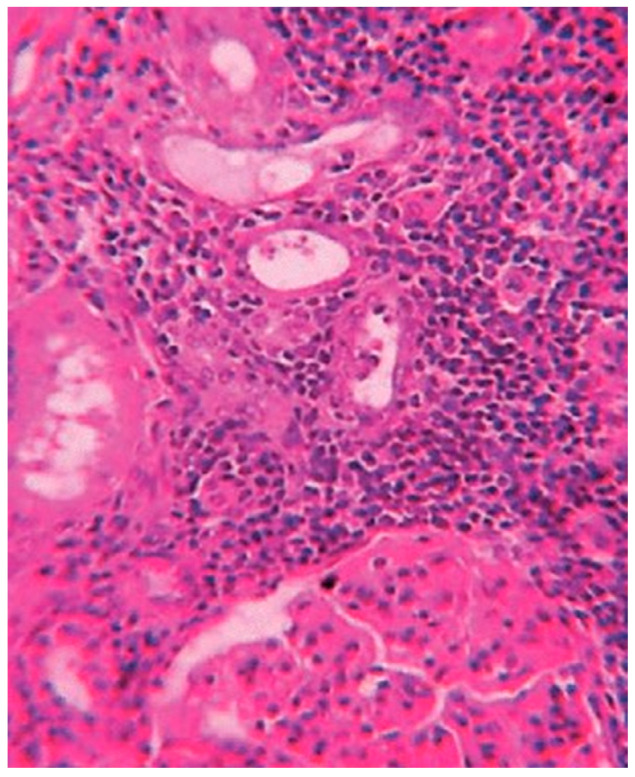
Tubular degeneration and necrosis of the kidney.

**Figure 3:**
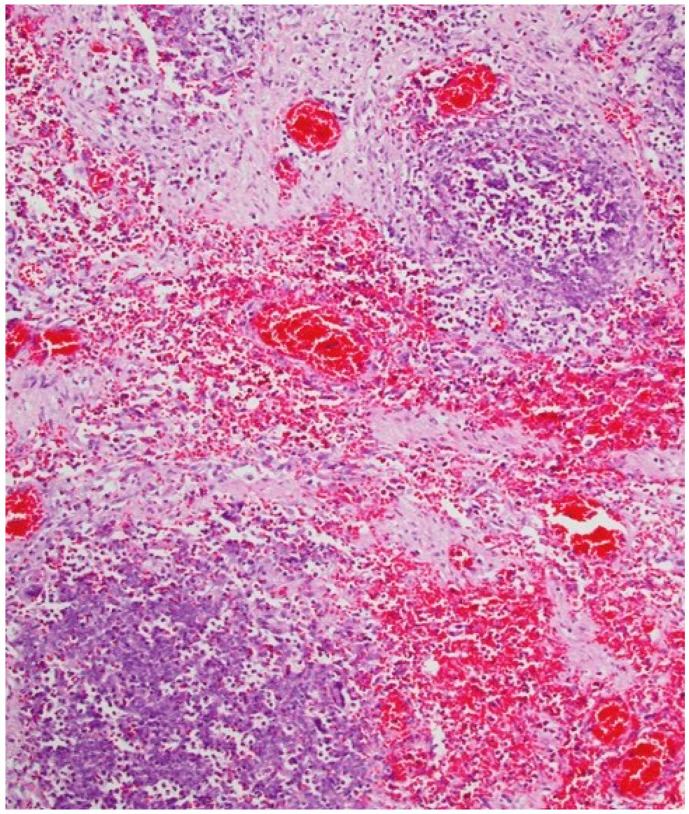
Mucosal inflammation and necrotic areas of the stomach.

Histological exams showed a degenerative myocardiopathy and segmental vascular insufficiency, associated with myocardial micronecrosis foci. Lungs presented diffuse alveolar damage, with chronic interstitial pulmonary disease. Kidney showed necrotic degenerative changes of the tubules and glomeruli with interstitial nephritis (see Figure 2). The presence of the greenish liquid was confirmed in the stomach and in the small intestine, with both presenting mucosal inflammation and gastric necrotic areas (see Figure 3). Finally, there was mild fatty liver disease.

### 3.3. Toxicological Analyses

The standard addition approach is suitably used as a quantification procedure when a blank matrix is not available. In the case presented here, the need to analyze autoptic samples such as gastric and colon contents was the main reason to choose this quantification procedure. Three aliquots of biological samples were added with three known diquat amounts, while the third was not spiked and was analyzed as a “zero” sample.

GC/MS-SIM analyses confirmed positivity to diquat in blood, urine, bile, and gastric and colon contents at the concentrations reported in Table 1.

### 3.4. Practitioners’ Work Analysis

The patient’s treatment procedure did not follow established guidelines or good clinical practices. Staff did not perform any clinical-diagnostical monitoring of the patient’s conditions, and this led to the lack of clarity about his clinical status. When the patient arrived at the E.R., his blood creatine kinase and myoglobin values were higher than normal, thus requiring careful clinical monitoring, further exams (i.e., echocardiogram), and more specialized evaluations in order to exclude possible lethal evolution linked to the evidenced muscular damage and to establish the possible consequences and origin of the evidenced abnormal parameters. The decision to simply prescribe a pain relief therapy without starting a close monitoring of the patient’s conditions cannot be endorsed. According to the guidelines, blood pressure, heart rate, respiratory rate, body temperature, and diuresis had to be strictly monitored, also to evidence the eventual onset of a more specific symptomatology.

## 4. Discussion

Diquat is a dipyridyl compound commonly used as a herbicide and structurally related to the commonly used paraquat. Diquat toxicity is a consequence of free oxygen radicals able to react with the cell membrane via lipid peroxidation; the final effect is cellular death and organ disfunction [17,18]. Reports on intoxication are usually related to suicidal ingestion, since its inhalation is not related to systemic toxicity (symptoms are normally reversible, with positive outcomes) [8,19]. After ingestion, specific symptoms can occur up to 48 h [5]. Due to its limited use, reports on diquat intoxication are few compared to those on paraquat. Tanen et al. reported 13 cases referred to diquat ingestion, with 9 of the 13 characterized by fatal outcomes [20]. Mortality rate was about 70%, with deaths related to gastrointestinal complications, pneumonia, paramedian pontine infarction, and renal failure [20]. In reviewing the literature on toxicity after diquat poisoning, Magalhães et al. [18] summarized the data since 1968, when the first man died from accidental oral absorption “of undiluted 20% formulation”. The authors schematized 57 cases, detailing the therapy administered and related effects: 30 of the 57 poisonings evolved into fatal outcomes, and death occurred from 5.5 h up to 1 month later [18]. As schematized by Magalhães et al., several analytical procedures are available for diquat analyses, involving different extraction/purification methods as well as detectors (colorimetric tests, UV-absorption, or mass spectrometric analysis). It must be stressed that obtaining an irrefutable result is mandatory in forensic toxicology, and consequently forensic determinations are almost entirely based on mass spectrometry.

Poisoning following diquat ingestion requires a timely and rapid diagnosis, since only supportive care therapies (often non-resolutive) are available.

Dipyridyl compounds present a wide distribution volume. Intestinal absorption is low, but organ and tissue uptake can reach lethal amounts within 6 to 18 h. Once distributed from blood to tissues, the toxicant is scarcely removed [21]. Usually, the absorbed dose plays a key role in determining the severity of intoxication or even death. The International Programme on Chemical Safety reports a lethal diquat dose of 6–12 g [22], with such amount fixed in 10 mL by the producers of a commercially available solution [23]. According to Schultz et al. [24], blood diquat concentrations in the range of (0.1–0.4) mg/L are associated with toxic effects; concentrations in the range (0.4–4.5) mg/L can result in coma/fatal outcomes. Literature data report fatal outcomes with less than 6 g of diquat; plasma concentrations of 0.5 mg/L within the first 24 h after ingestion are associated with systemic poisoning [25]. In the case presented here, toxicological analyses evidenced a blood diquat concentration of 1.2 mg/L, more than twice the minimum needed to observe a systemic poisoning. Moreover, given the diquat half-life and the estimated time between death and autopsy (in Italy, judicial autopsy cannot be performed before 24 h), it is more than reasonable to deduce that ante-mortem levels were even higher, and in line with the ingestion of a lethal dose. On his arrival at the E.R., the man declared a herbicide inhalation, without specifying which one, and complained of pain in his legs and feet; physicians performed general checks (blood analyses; ECG; chest, abdomen, and pelvis CT-angio with contrast agent). The clinical picture was normal, except for some values, which were attributed to a general nonspecific inflammation and a respiratory alkalosis, probably due to hypoxia or pulmonary hyperventilation. This hypothesis was in line with the patient’s declarations. Physicians transferred the man in the Short-Stay Observation and gave him antipyretic/analgesic (paracetamol) therapy and hydration.

Yu et al. [26] studied three cases of acute diquat poisoning with resulting encephalopathy. The data highlighted renal failure, neurological disorders, and respiratory failure following ingestion of 50–100 mL of a 20 g/100 mL diquat formulation; blood diquat concentrations were determined in two out of three cases, as 0.43 µg/mL and 0.93 µg/mL. One of the patients died after 18 days of hospitalization due to cardiac arrest. The second patient still presented dystasia and trouble walking three months after the adverse event; the last one had nearly total symptom relief after 57 days.

Hanston et al. [27] published results obtained in a case of suicide by ingestion of about 300 mL of 20% diquat solution (corresponding to about 60 g). The man arrived at the E.R. 4 h after the poisoning, presenting neurological disorders and progressive anuria (after 14 h, he became anuric). Gastric lavage and treatment with active charcoal were performed. The serum diquat concentration was 64 µg/mL. His hemodynamic status worsened within 22 h from diquat ingestion, and he died from refractory cardiocirculatory collapse 26 h after the poisoning. At autopsy, the brain presented abnormalities probably due to status epilepticus, although not specific for diquat poisoning; abundant necrotic lesions characterized renal tubules, and fibrin deposits were present in the glomeruli; the pancreas had signs of necrosis; lung and myocardium showed interstitial oedema [27]. Post-mortem toxicological analyses performed on organs evidenced higher diquat concentrations in the kidney (4.5 µg/g tissue), followed by lung (3.4 µg/g tissue), liver (2.3 µg/g tissue), brain (1.6 µg/g tissue), and heart (1.1 µg/g tissue) [27].

After a correct diagnosis of diquat ingestion, a prompt gastrointestinal decontamination can reduce/prevent the absorption [4,10]. Adsorbent agents such as bentonite (7.5% suspension) and Fuller earth (15% suspension) are useful, and if not available, active carbon can be of help up to one hour after the ingestion (beyond that time, use of active carbon requires special care to avoid bleeding, perforations, or injuries due to additional trauma on already traumatized tissues) [11,18]. No literature studies support the efficacy of active carbon-based treatments to avoid death. Five out of seven cases for which gastrolus was immediately performed resulted in a fatal outcome within 1–7 days; the other two patients ingested low diquat amounts (5 mL), and in one case, such an amount was fatal after seven days [10,28,29,30,31,32].

Post-mortem toxicological analyses performed in the case discussed here evidenced positivity in all specimens (83.1 mg/L in gastric content, 6.3 mg/L in colon content, 106.3 mg/L in bile, and 0.03 mg/L in urine). Gastric and colon content positivities, with concentrations higher than urinary results, attested to the analyte accumulation in gastrointestinal fluids. According to data from Crabtree et al. [33] showing such a mechanism develops rapidly within 24 h from absorption, it is reasonable to assume that the patient ingested diquat long before 8 p.m. of the first day of E.R. admittance. As in other diquat intoxication reports [10,20,34], the patient presented non-specific symptoms; was vigilant, without any discomfort; and his lips, tongue, and gums were not burned. Moreover, there was no airway oedema, and chest X-ray evidenced no infiltration. Very often in literature reports, family members or the patient themselves declares the ingestion of the pesticide upon arrival at the hospital, thus facilitating a correct diagnosis. Despite this, a fatal outcome occurred in 70% of the cases. In the case presented here, the patient declared a herbicide inhalation, without specifying the exact compounds or commercial formulation. Data from the gastric contents confuted this, since ingestion was the most reasonable absorption method.

Healthcare professionals are required to comply with the rules of conduct and good practices defined in specific guidelines. In Italy, such recommendations are mandatory when guidelines are validated by the Ministry of Health [35]. Among others, physicians are asked to correctly draw up and archive health records for a prescribed time [36]. They must take the necessary precautions to avoid the onset of complications for the patients [37] and inform them about the health treatment and its foreseeable consequences [38,39].

The most frequent source of errors in the E.R. is related to the definition of the colour code assigned during the triage and the diagnosis process. Regardless of the possible exposure to chemicals, decisional flow-charts are available to help to choose the triage code, also indicating the most appropriate analyses (laboratory and instrumental) and the most pertinent therapy [40]. According to the Italian guidelines for Short-Stay Observation [41], hospitalization is appropriate with altered state of consciousness, persistent altered vital functions, and foreseeable late toxicity. When the results of laboratory tests are within normal parameters and symptoms subside in 4–6 h, most patients can be discharged. If a voluntary chemical ingestion is reasonable, a psychiatric evaluation could be necessary. A correct treatment of intoxications in adults must include clinical and laboratory investigations as well as a diagnostic analysis of the patient in order to define the exact chemical absorbed and establish a general and specific therapy [41].

Acute intoxication can derive from accidental ingestion, injection, inhalation, or body exposure (through skin, eyes, and mucous membranes), mostly occurring for children and older people (as consequence of an altered mental status or visual disturbances) or because of the precise suicidal intent of the subject. The collection of anamneses can be of great utility to define both the chemicals involved and the absorption route. Clinical examination must highlight any alterations in vital functions, through clinical monitoring of breathing (airway patency, ventilation), circulation (PA, cardiac arrhythmias), and central nervous system (convulsions, coma).

Serum creatine phosphokinase (SCK), whose concentration reflects the extent of acute muscle necrosis, is considered a predictive index, as it can be used to assess the severity of poisoning [42,43,44,45]. Damage to muscle tissues is reported for dipyridyls intoxications [46]. Monitoring creatine phosphokinase is useful to predict the patient’s prognosis, since an increase in serum values can act as an alarm signal to start an intensive monitoring. Instrumental investigations (electrocardiogram, x-ray of the chest and abdomen, esophagogastroduodenoscopy) can provide additional information that is useful for diagnostic and therapeutic purposes.

Once ingested, treatment of diquat poisoning includes skin and eye decontamination (with copious amounts of water in the case of skin contact) and gastrointestinal decontamination with adsorbents (with bentonite, Fuller’s earth, or activated carbon). The effectiveness of gastric lavage in diquat poisoning has not been proven; it should not be performed later than one hour after ingestion, to avoid the risk of bleeding, perforation, or injury due to additional trauma to already traumatized tissues. Pain derived from the deep erosion of the mucous membranes of the digestive tract may require the use of morphine; mouthwashes, cold liquids, ice cream, or anaesthetic can help relieve pain in the mouth and throat. It is essential to maintain adequate diuresis by fluid infusion (physiological solution, ringer acetate, 5% glucose). Such therapy is extremely advantageous in the early stages of intoxication to correct dehydration and accelerate the elimination of the toxin. A careful monitoring of fluid balance allows prevention of fluid overload if renal failure develops. If kidney failure occurs, the intravenous infusion of liquids must be stopped, and haemodialysis is recommended, although it is not effective in purifying blood and tissues from the diquat. Oxygen should be administered only when the patient develops severe hypoxemia; high concentrations of oxygen in the lungs may increase the extent of damage induced by diquat [11]. In severe poisoning, treatment must be guaranteed in the intensive care unit (IUC), to allow appropriate monitoring of vital functions and for invasive medical procedures.

If diquat has spread to the tissues, procedures and treatment to remove the toxin from the blood are insufficient.

In the clinical case presented, the behaviour of Intensive Brief Observation (OBI) department doctors was considered incorrect due to the omission of clinical and laboratory monitoring. Such improper conduct prevented the assessment of the foreseeable worsening of the clinical conditions. The incorrect conduct resulted in a loss of the patient’s chance in terms of survival. Death was not avoidable with certainty, as diquat is very toxic, and the decontaminating treatment has limited efficacy (the patient also suffered from cardiovascular comorbidities).

The misconduct of the physicians was judged to be the cause of damages in a civil action, while it was deemed to have no consequences in penal trial. This difference relates to the different criteria of conviction for professional liability in civil and criminal law: in the civil court, the causal relationship is recognized if the misconduct has a greater probability than other possible causes to produce damages to the psycho-physical integrity of the person, whereas in criminal proceedings, the causal link must be demonstrated “beyond any reasonable doubt” (degree of probability close to certainty). Moreover, in the civil judgement, loss of survival chance and/or worsening of life quality are considered among possible personal injuries [47].

## 5. Conclusions

The clinical management of the subject poisoned with diquat (and, more generally, of a person who is intoxicated by any chemical) is quite complex and requires great experience. In addition to a correct initial diagnostic framework, it also requires careful clinical and laboratory monitoring; in fact, acute intoxication is a dynamic process that can quickly worsen and lead to lethal complications, although onset symptoms may be blurred.

After diquat poisoning, SCK is a valid biological parameter to evaluate the severity of the intoxication, and its monitoring can give prognostic indications.

## Figures and Tables

**Table 1:** Diquat concentrations evidenced by GC/MS analyses performed on post-mortem blood, urine, bile, and gastric and colon contents.

Matrix	Diquat (mg/L)
blood	1.2
bile	106.3
urine	0.03
gastric content	83.1
colon content	6.3

## Data Availability

Not applicable.
